# Expression of CXCR4 on T-cell subsets and Plasma IL-17 Concentrations in Patients with Aplastic Anaemia

**DOI:** 10.1038/s41598-017-08699-z

**Published:** 2017-08-22

**Authors:** Qian Niu, Qiang Zhou, Yumei Liu, Hong Jiang

**Affiliations:** Department of Laboratory Medicine, West China Hospital, Sichuan University, Chengdu, The People’s Republic of China

## Abstract

Acquired aplastic anaemia (AA) is caused by T-cells migrating to and attacking bone marrow (BM) in response to chemokines (e.g., CXCR4). We investigated CXCR4 expressions on circulating T-cell subsets, plasma IL-17A concentrations, and their correlations with AA manifestations. We enrolled 71 patients with acquired AA (36 severe AA cases [SAA] and 35 non-severe AA cases [NSAA]) and 42 healthy volunteers. We used flow cytometry and ELISA to measure circulating CD4^+^ and CD8^+^ T-cells, their CXCR4 expressions, and plasma IL-17A concentrations. Compared to the healthy controls, SAA patients had fewer peripheral CD4^+^ T-cells, more CD8^+^ T-cells, and a significantly decreased CD4^+^/CD8^+^ ratio which was positively correlated with AA manifestations. Patients with SAA or NSAA had higher proportions of CD4^+^CXCR4^+^ and CD8^+^CXCR4^+^ T-cells, which were negatively correlated with haemoglobin concentrations and absolute neutrophil counts. Patients with SAA or NSAA had higher plasma IL-17A concentrations, which were negatively correlated with AA manifestations and the CD4^+^/CD8^+^ ratio. IL-17A concentrations showed a very week correlation with CD4^+^CXCR4^+^ T-cells frequencies, and no correlation with CD8^+^CXCR4^+^ T-cells frequencies. Aberrant CXCR4 expression may allow circulating T-cells, especially CD8^+^ T-cells, to infiltrate BM during AA progression. Elevated IL-17A concentrations may contribute to AA progression outside of the CXCR4-SDF-1α axis.

## Introduction

Aplastic anaemia (AA) is a syndrome that is characterized by bone marrow (BM) aplasia and failure, as well as peripheral blood pancytopenia. Most AA cases are acquired, idiopathic, and can occur in both children and adults. Acquired AA (aAA) is considered an immune-mediated disease, which is supported by the fact that approximately 80% of patients with aAA respond to immunosuppressive therapy using anti-thymocyte globulin and cyclosporin^1^. The BM destruction in untreated cases is the result of an abnormal expansion of helper T-cells (Th1, Th2, and Th17 cells) and the decreased or skewed immunophenotype and function of regulatory T-cells^2–5^. However, the proportion of mature CD4^+^ and CD8^+^ T-cells in BM is very small, which suggests that dysregulated T-cells must be sequestered to the BM to exert their pathogenic effects. In this context, the interactions between chemokine receptors and their ligands play important roles in mediating T-cell migration. For example, CXCR4 is a chemokine receptor that is expressed on T-cells and facilitates their migration toward its natural ligand (stromal-cell derived factor-1α [SDF-1α]), which is strongly expressed by BM stromal cells^6–8^.

Dysregulated expression of CXCR4/SDF-1α is also associated with the pathology of various autoimmune diseases, including rheumatoid arthritis, systemic lupus erythematosus, and multiple sclerosis^9–11^. In 2015, Arieta *et al*.^12^ were the first to report that CXCR4 expression on pathogenic T-cells facilitates their BM infiltration in a mouse model of AA. However, the contribution of CXCR4 to the pathology of human aAA remains unclear.

IL-17 is a well-known pro-inflammatory cytokine that is mainly produced by Th17 cells, and has emerged as a critical factor that enhances metastasis in breast cancer through the CXCR4/SDF-1α pathway^13, 14^. Patients with aAA express both IL-17 and Th17 cells, which suggests that they may play a role in the disease’s pathogenesis^15–17^. However, it remains unclear whether IL-17 mediates the sequestration of pathogenic T-cells to the BM through the CXCR4/SDF-1α axis.

The present study aimed to investigate the expression of CXCR4 on peripheral blood CD4^+^ and CD8^+^ T-cells, the plasma concentration of IL-17A, and their relationships in patients with aAA. Our results suggest that enhanced CXCR4 expression on CD4^+^ T-cells was positively correlated with increased plasma IL-17A concentrations in patients with AA. In addition, the level of CXCR4 expression on T-cells and IL-17A concentration were both positively correlated with AA severity.

## Methods

This prospective study was performed in accordance with a protocol that was approved by the Ethics Committees of the Chinese Human Genome Project and the West China Hospital. All participants provided their written informed consent.

## Participants

We enrolled 42 healthy volunteers (19 men and 23 women) and 71 patients with newly diagnosed aAA (32 men and 39 women) from the Hematology Department of the West China Hospital (Sichuan University) between July 2015 and April 2016. All patients were diagnosed and classified based on the Guidelines for the Diagnosis and Management of Aplastic Anemia^18^. The disease was considered severe AA (SAA) if the patient had pancytopenia with ≥2 of the following parameters: a neutrophil count of <0.5 × 10^9^/L, a platelet count of <20 × 10^9^/L, and a reticulocyte count of <20 × 10^9^/L with hypocellular bone marrow. We excluded patients with congenital AA and other haematological or autoimmune diseases, as well as patients with infections [including hepatitis B virus (HBV), hepatitis C virus (HCV), human immunodeficiency virus (HIV), Epstein-Barr virus (EBV) and tuberculosis (TB) infection]. The characteristics of the patients and healthy controls are shown in Table 1.

**Table 1:** Characteristics of the patients with aplastic anaemia and the healthy controls.

Characteristics	SAA (N = 36)	NSAA (N = 35)	Healthy controls (N = 42)
Age (year)	31.9 (14.9)	34.1 (15.2)	33.9 (9.4)
Sex			
Male	17 (47.2)	15 (42.9)	19 (45.2)
Female	19 (52.8)	20 (57.1)	23 (54.8)
Haematological parameters			
Haemoglobin (g/L)	67.8 (15.5)	81.7 (23.5)	144.9 (26.7)
Platelet count (×10^9^/L)	15.5 (8.8)	28.1 (11.3)	211.2 (51.9)
Absolute neutrophil count (×10^9^/L)	0.23 (0.09)	1.31 (0.52)	3.48 (0.75)
Absolute lymphocyte count (×10^9^/L)	1.01 (0.31)	1.37 (0.50)	1.92 (0.36)

Data are presented as mean (standard deviation) for most variables, although sex is presented as *n* (%).

SAA: severe aplastic anaemia; NSAA: non-severe aplastic anaemia.

## Blood samples

All participants provided a 3-ml fasting blood sample, which was collected into a BD Vacutainer tube containing sodium heparin at 8:00–9:00 AM. The whole blood was used for flow cytometry. Plasma was obtained after centrifugation and stored at −80 °C for the cytokine testing.

## Flow cytometry

The flow cytometry was performed after incubating 50 μL of whole blood with monoclonal antibodies for 30 min at 4 °C. The monoclonal antibodies targeted human CD3 (clone SK7, PerCP-Cy5-5), CD4 (clone RPA-T4, FITC), CD8 (clone SK2, PE), and CXCR4 (CD184, clone 12G5, APC), and were all from BD Biosciences (San Diego, USA). Isotype controls were given to enable correct compensation and confirm antibody specificity. Stained cells were run on a FACS Canto cytometer (BD Bioscience), and the data were analysed using FACSDiva software (BD Bioscience).

## Enzyme-linked immunosorbent assay (ELISA)

The IL-17A level was determined using a specific human IL-17A Platinum ELISA kit (Cat#BMS2016; Bender Med Systems, Burlingame, USA). The limit and sensitivity of detection for the ELISA kit are 1.6–100 pg/ml and 0.5 pg/ml, respectively. Protocol recommended by manufacturer was followed. All samples were measured in duplicate. Results are expressed as pg/ml.

## Statistical analysis

Summary statistics (number and percentage or median and interquartile range [IQR]) were used to describe the participants’ baseline characteristics. Numerical results were analysed using the IBM SPSS software (version 20.0; IBM Corp., Armonk, NY). The significance level was set at 5% for all statistical tests. The data were initially analysed using analysis of variance or the Kruskal-Wallis H test. If a significant result was observed, the Student-Newman-Keuls or Mann-Whitney tests were used to detect inter-group differences. Spearman’s correlation coefficient was used to test the correlations between pairs of two continuous variables.

## Results

### Frequencies of circulating T-cell subsets in patients with AA and healthy controls

**Figure 1:**
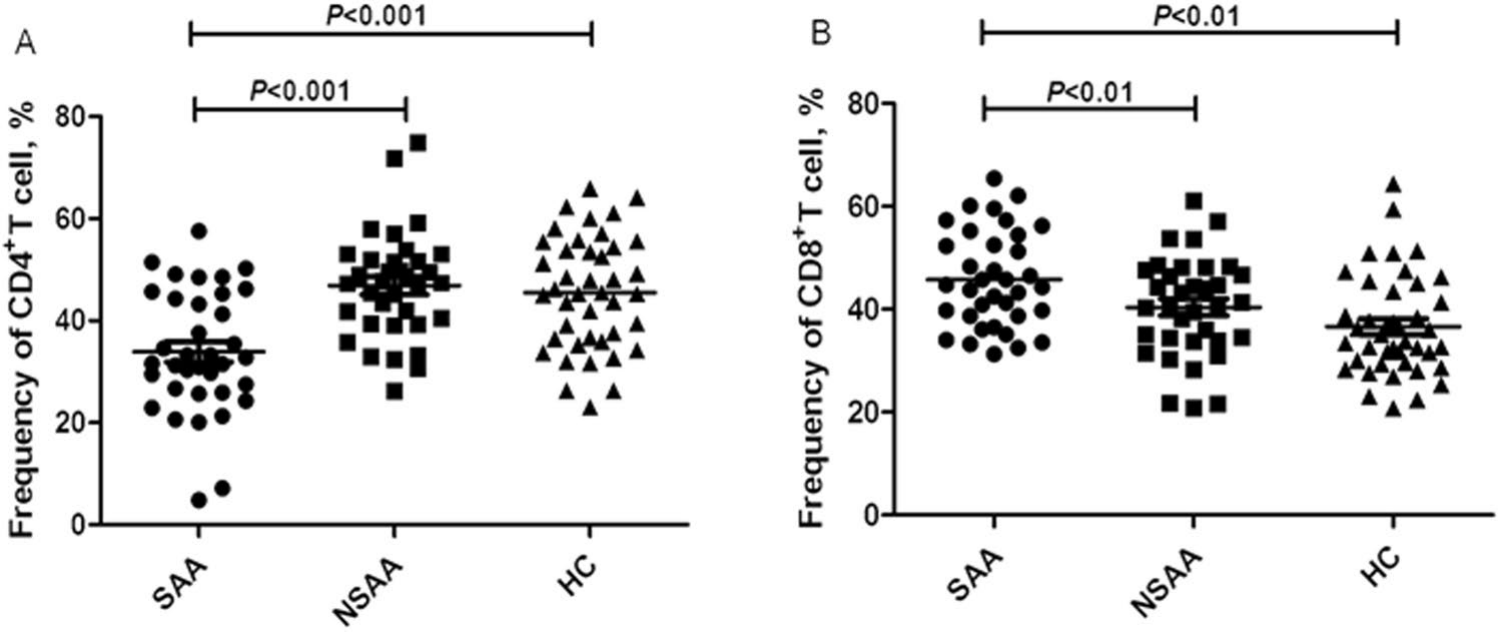
Frequencies of circulating T-cell subsets in patients with aplastic anaemia and the healthy controls. SAA: severe aplastic anaemia; NSAA: non-severe aplastic anaemia; HC: healthy control. (**A**) Frequencies of CD4^+^ T-cells in patients with SAA (•), patients with NSAA (■), and the HC (▲). (**B**) Frequencies of CD8^+^ T-cells in patients with SAA (•), patients with NSAA (■), and the HC (▲).

**Figure 2:**
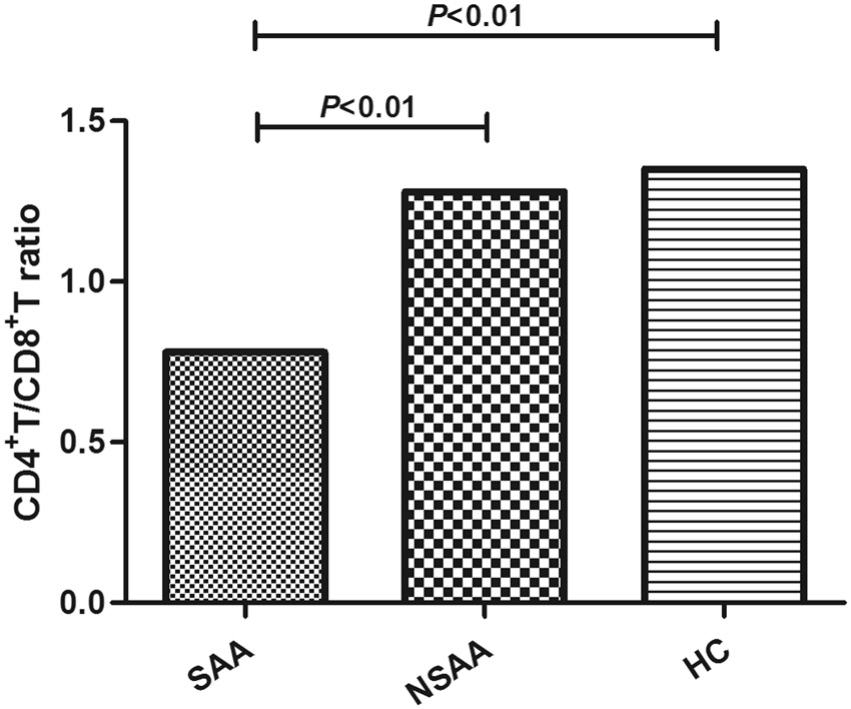
Circulating CD4^+^/CD8^+^ T-cell ratios in patients with aplastic anaemia and the healthy controls. SAA: severe aplastic anaemia; NSAA: non-severe aplastic anaemia; HC: healthy control.

The frequency of peripheral CD4^+^ T-cells was significantly lower in patients with SAA (33.89 ± 12.04%), compared to patients with NSAA (46.87 ± 10.43%) and the healthy controls (45.50 ± 11.04%) (*P* < 0.001, Fig. 1A). However, the frequency of peripheral CD8^+^ T-cells was also significantly higher in patients with SAA (45.77 ± 9.38%), compared to patients with NSAA (40.39 ± 9.73%) and the healthy controls (36.64 ± 9.77%) (*P* < 0.01, Fig. 1B). This resulted in a significantly lower CD4^+^/CD8^+^ T-cell ratio in the SAA group (0.78 ± 0.33), compared to the NSAA group (1.28 ± 0.64) and the control group (1.35 ± 0.53) (*P* < 0.01, Fig. 2).

### Frequencies of circulating CXCR4^+^T-cell subsets in patients with AA and healthy controls

**Figure 3:**
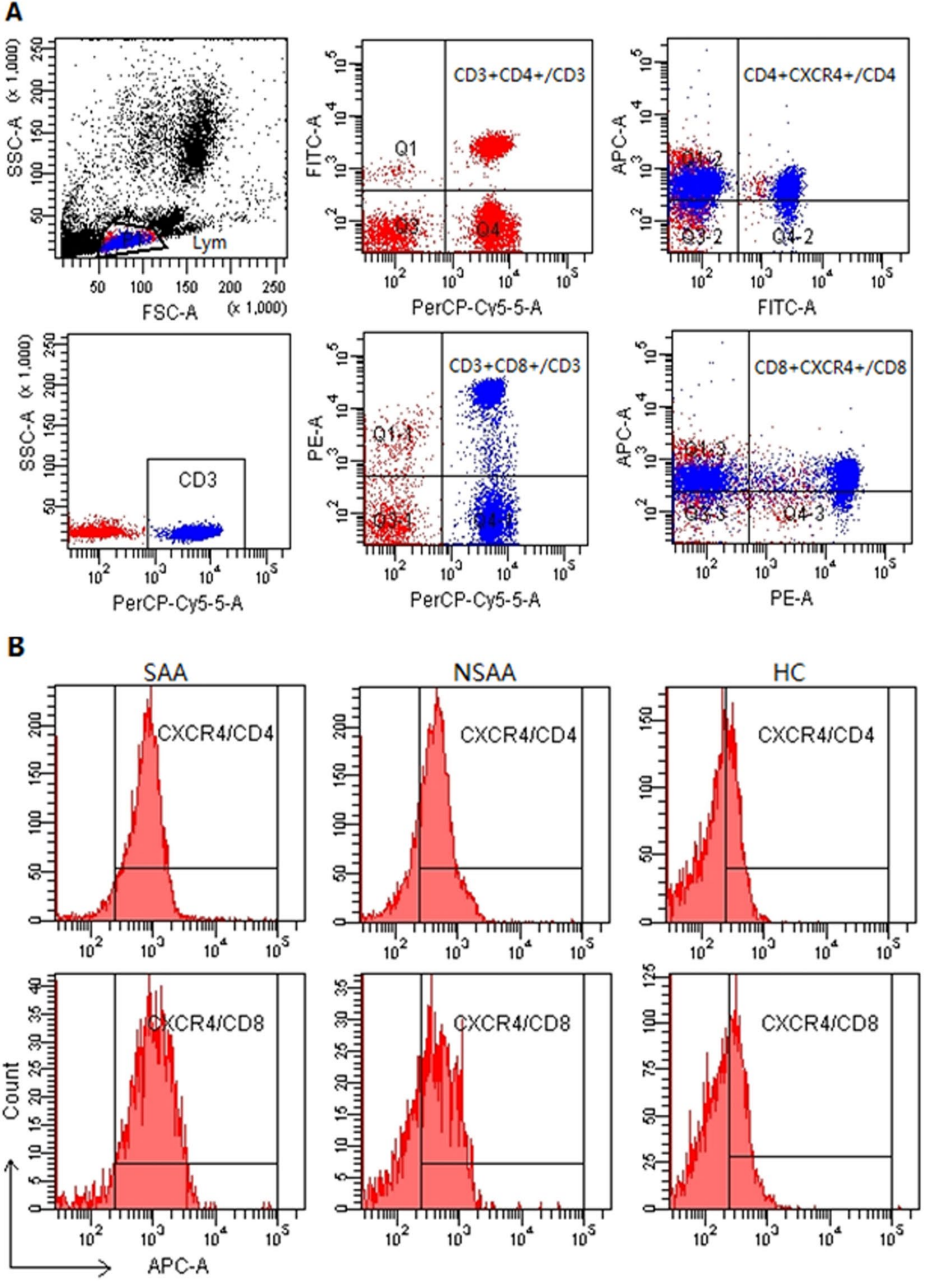
Flow cytometry of CXCR4 expression on CD4^+^ and CD8^+^ T-cells. (**A**) Scatter plots of flow cytometry. Gating on the CD3^+^ T-cells, the frequencies of CD4^+^ and CD8^+^ T-cells were calculated. Gating on the CD4^+^ T-cells and the CD8^+^ T-cells allowed us to calculate the frequencies of CD4^+^CXCR4^+^ T-cells and CD8^+^CXCR4^+^ T-cells, respectively. (**B**) Representative histograms of CXCR4 expression on CD4^+^ and CD8^+^ T-cells for three groups. SAA: severe aplastic anaemia; NSAA: non-severe aplastic anaemia; HC: healthy control.

**Figure 4:**
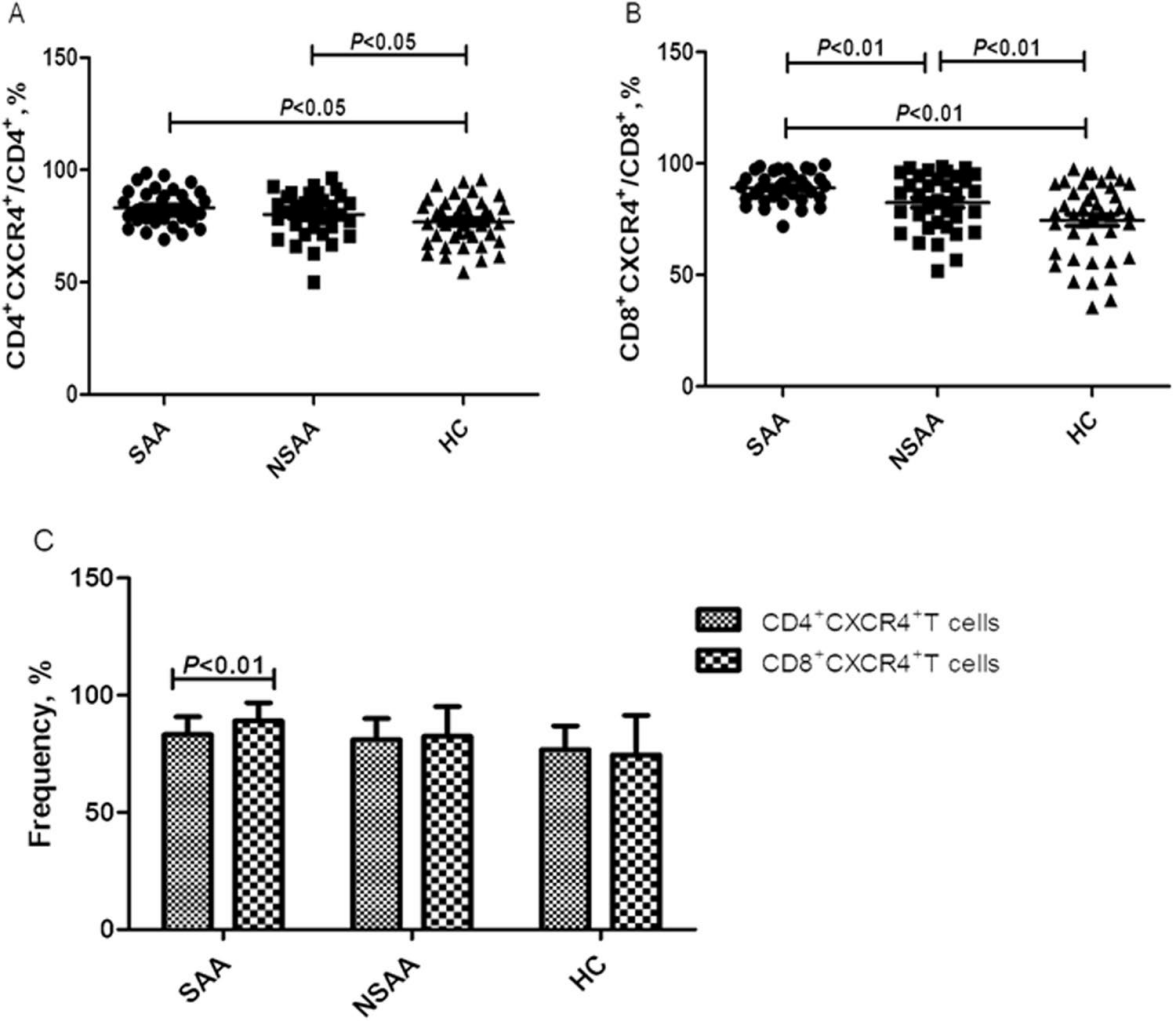
Frequencies of circulating CXCR4^+^ T-cell subsets in patients with aplastic anaemia and the healthy controls. SAA: severe aplastic anaemia; NSAA: non-severe aplastic anaemia; HC: healthy control. (**A**) Frequencies of CD4^+^CXCR4^+^ T-cells (gating on CD4^+^ T-cells) in patients with SAA (•), patients with NSAA (■), and the HC (▲). (**B**) Frequencies of CD8^+^CXCR4^+^ T-cells (gating on CD8^+^ T-cells) in patients with SAA (•), patients with NSAA (■), and the HC (▲). (**C**) Intra-group comparisons of the frequencies of CD4^+^CXCR4^+^ T-cells and CD8^+^CXCR4^+^ T-cells.

Gating on the CD4^+^ or CD8^+^ T-cells allowed us to calculate the frequencies of circulating CD4^+^CXCR4^+^ or CD8^+^CXCR4^+^ T-cells (Fig. 3A). Figure 3B showed the representative flow cytometric histograms of CXCR4 expression on CD4^+^ and CD8^+^ T-cells for three groups. As shown in Fig. 4A, the frequency of CD4^+^CXCR4^+^ T-cells was significantly higher in the patients with SAA (83.2 ± 7.6%) and NSAA (81.0 ± 9.1%), compared to the healthy controls (76.8 ± 10.1%) (*P* < 0.05). However, there was no significant difference between the two AA groups (*P* > 0.05). Significant differences in the frequencies of CD8^+^CXCR4^+^T-cells were observed when we compared all three study groups (*P* < 0.01, Fig. 4B). The SAA group had the highest frequency of CD8^+^CXCR4^+^ T-cells (89.1 ± 7.7%), which was followed by that in the NSAA group (82.4 ± 12.8%) and the control group (74.6 ± 16.8%).

We also performed intra-group comparisons of the frequencies of CD4^+^CXCR4^+^ and CD8^+^CXCR4^+^ T-cells. The only significant difference was observed in the SAA group, which had a significantly lower frequency of CD4^+^CXCR4^+^ T-cells, compared to CD8^+^CXCR4^+^ T-cells (*P* < 0.01, Fig. 4C). The NSAA group had a slightly lower frequency of CD4^+^CXCR4^+^ T-cells, compared to the CD8^+^CXCR4^+^ T-cells in the NSAA group and the CD8^+^CXCR4^+^ T-cells in the control group, although these differences were not statistically significant (*P* > 0.05, Fig. 4C).

### Plasma concentrations of IL-17A in patients with AA and the healthy controls

**Figure 5:**
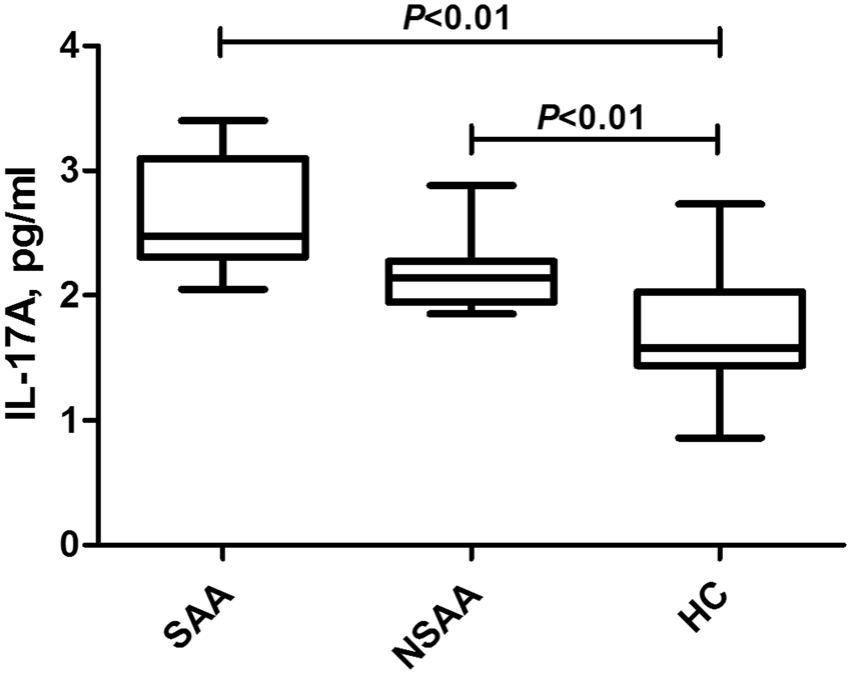
Plasma concentrations of IL-17A in patients with aplastic anaemia and the healthy controls. SAA: severe aplastic anaemia; NSAA: non-severe aplastic anaemia; HC: healthy control.

Compared to the control group (IL-17A: 1.58 pg/ml [IQR: 1.44–2.03 pg/ml]), the plasma concentrations of IL-17A were significantly higher in the SAA group (2.47 pg/ml [IQR: 2.31–3.10 pg/ml]) and the NSAA group (2.12 pg/ml [IQR: 1.95–2.28 pg/ml]) (both *P* < 0.01). However, there was no significant difference when we compared the SAA and NSAA groups (*P* > 0.05) (Fig. 5).

### Correlation analysis

**Figure 6:**
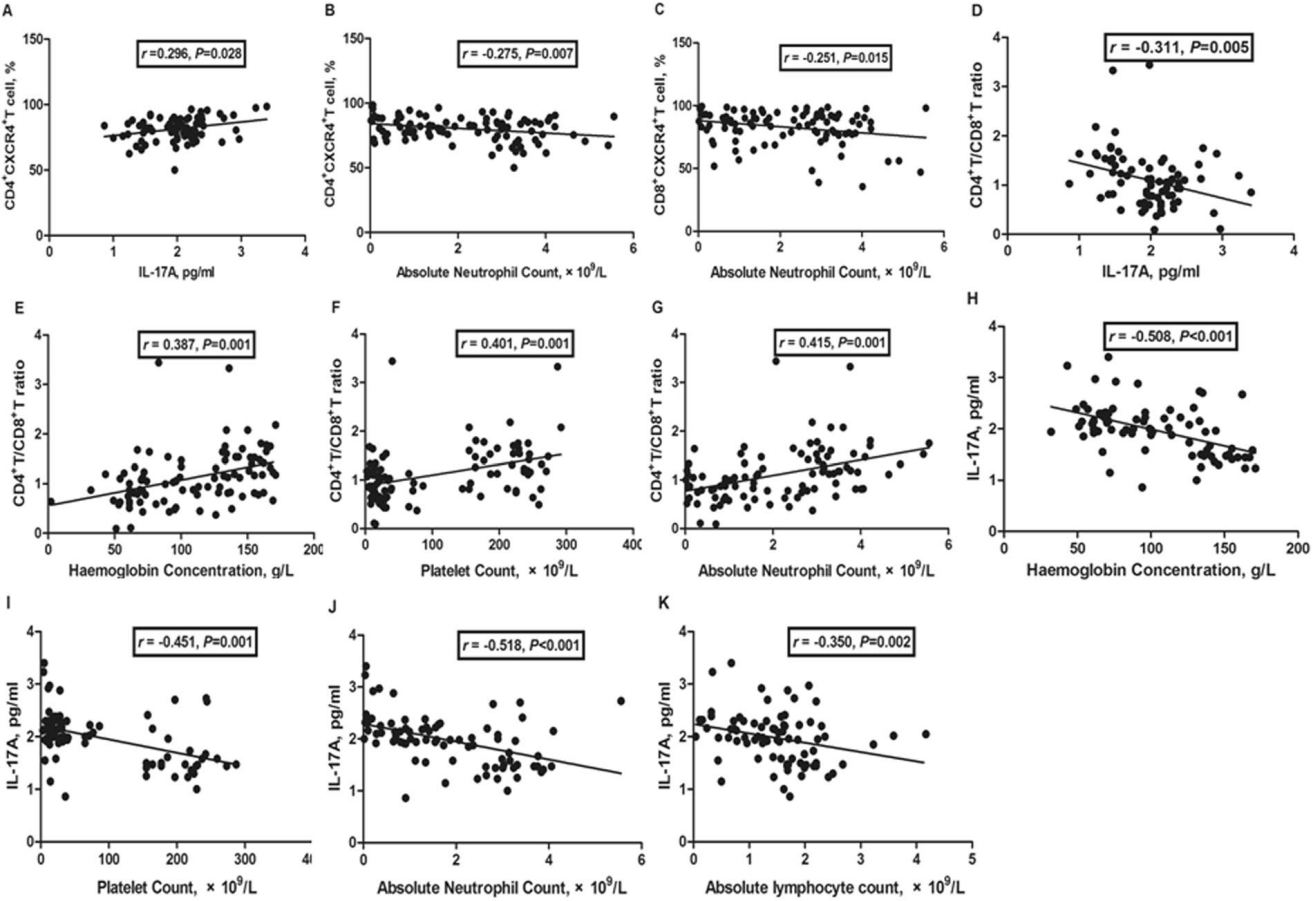
Correlation analysis. (**A**) The percentage of CD4^+^CXCR4^+^ T-cells was positively correlated with plasma IL-17A concentrations; and (**B**) negatively correlated with absolute neutrophil counts; (**C**) The percentage of CD8^+^CXCR4^+^ T-cells was negatively correlated with absolute neutrophil counts; (**D**) The CD4^+^/CD8^+^ T-cell ratio was negatively correlated with plasma IL-17A concentrations; (**E**) The CD4^+^/CD8^+^ T-cell ratio was positively correlated with haemoglobin concentrations, (**F**) platelet counts and (**G**) absolute neutrophil counts. (H) IL-17A concentrations were negatively correlated with haemoglobin concentrations, (**I**) platelet counts, (J) absolute neutrophil counts and (K) absolute lymphocyte counts.

The percentage of CD4^+^CXCR4^+^T-cells was positively correlated with plasma IL-17A concentrations (*r* = 0.296, *P* = 0.028, Fig. 6A), but was negatively correlated with absolute neutrophil counts (*r* = –0.275, *P* = 0.007, Fig. 6B). The percentage of CD8^+^CXCR4^+^ T-cells was negatively correlated with absolute neutrophil counts (*r* = −0.251, *P* = 0.015, Fig. 6C). The CD4^+^/CD8^+^ T-cell ratio was negatively correlated with plasma IL-17A concentrations (*r* = −0.311, *P* = 0.005, Fig. 6D), but was positively correlated with haemoglobin concentrations (*r* = 0.387, *P* = 0.001, Fig. 6E), platelet counts (*r* = 0.401, *P* = 0.001, Fig. 6F), and absolute neutrophil counts (*r* = 0.415, *P* = 0.001, Fig. 6G). IL-17A concentrations were negatively correlated with haemoglobin concentrations (*r* = −0.508, *P* < 0.001, Fig. 6H), platelet counts (*r* = −0.451, *P* = 0.001, Fig. 6I), absolute neutrophil counts (*r* = −0.518, *P* < 0.001, Fig. 6J), and absolute lymphocyte counts (*r* = −0.35, *P* = 0.002, Fig. 6K).

## Discussion

AA is primarily driven by the aberrant regulation of CD8^+^ T-cells and CD4^+^ Th1 cells, which facilitates T-cell sequestration to the BM and its subsequent destruction. In patients with aAA, activated circulating CD8^+^ T-cells are a lymphocyte subset that inhibit hematopoiesis^19^, and 28% of patients with AA have an elevated percentage of activated CD8^+^ T-cells in their BM^20^. In the present study, patients with severe AA had a significantly lower CD4^+^/CD8^+^ T-cell ratio, which was the result of fewer CD4^+^ T-cells and more CD8^+^ T-cells. Furthermore, the ratio decreased with disease severity and was positively correlated with the patients’ manifestations (including haemoglobin concentrations, platelet counts, and absolute neutrophil counts). These findings support the potentially critical role of CD8^+^ T-cells in the pathogenesis of aAA.

The mechanisms that govern the sequestration of pathogenic T-cells to the BM remain unclear. However, the CXCR4 chemokine receptor facilitates cellular chemotaxis in response to its natural ligand (SDF-1α), which is strongly expressed in the BM^8, 21^. Aberrantly elevated CXCR4 concentrations have been reported in numerous autoimmune conditions, and may provide a means for malignant cells to target SDF-1α-enriched sites, such as the BM^14–17, 22^. The only study to demonstrate CXCR4’s critical role in facilitating pathogenic T-cells homing to the BM was performed in a mouse model of AA^17^, and data from patients with AA remain scarce. However, we used flow cytometry to evaluate circulating CD4^+^ and CD8^+^ T-cells from patients with aAA, and observed that high CXCR4 expression was associated with disease severity. These findings suggest that the interaction between CXCR4 and SDF-1α may provide a mechanism for sequestering pathogenic T-cells to the BM during AA progression. In addition, the percentage of CD8^+^CXCR4^+^ T-cells was significantly higher than that of CD4^+^CXCR4^+^ T-cells in patients with SAA, and was negatively correlated with absolute neutrophil counts. These findings suggest that CD8^+^CXCR4^+^ T-cells may also play a critical role in AA exacerbation, which is indirectly supported by previous findings of high CXCR4 expression on BM-infiltrating CD8^+^ T-cells.

Because IL-17A is a pro-inflammatory cytokine, its dysregulation is associated with various autoimmune disorders, such as type 1 diabetes, rheumatoid arthritis, and Sjögren’s syndrome^23–25^. Furthermore, our data support previous findings that patients with active AA had elevated IL-17 concentrations^15, 16^, and suggest that IL-17 may also play a role in AA progression. Further analyses revealed a moderate negative correlation between plasma IL-17A concentration and the CD4^+^/CD8^+^ T-cell ratio, and negative correlation between IL-17A concentrations and the patients’ clinical manifestations of AA. However, the correlation between IL-17A concentrations and CXCR4 expressions on circulating CD4^+^ or CD8^+^ T-cells was very weak or absent. When considered together, these results suggest that IL-17A may contribute to AA progression outside of the CXCR4-SDF-1α axis.

However, further studies of BM from patients with AA are needed to validate our findings. Actually, evaluating the role of CXCR4 and IL-17A on outcome after therapy, which need more evidences from further BM assessment and prospective analyses, is of great value in assessing efficacy of clinical management of AA. In addition, CCR4^+^CCR6^+^ T cells were recently validated to produce IL-17 and expressed the Th17-specific transcription factor RORC, establishing a link between CD4^+^ T cell trafficking potential and immunologic function^26^. The co-expression of CCR6 in AA patients can be of interest. And, all these will be our future research contents and directions. To elucidate potential therapeutic approaches, these studies should evaluate the full array of chemokine receptors and adhesion molecules that can facilitate the sequestration of pathogenic CD4^+^ and CD8^+^ T-cells to the BM during AA progression.

## Conclusions

Ours is the first study to reveal that aberrant CXCR4 expression may help sequester circulating T-cells, and especially CD8^+^ T-cells, to the BM during aAA progression. Elevated IL-17A concentrations may also help mediate the abnormal CD4^+^/CD8^+^ T-cell ratio, but were not significantly associated with CXCR4 expression.
